# Diphtheria at Public Schools

**Published:** 1890-11-15

**Authors:** 


### The Hospital

A Weekly Journal of

Science, Medicine, Nursing, and Philanthropy.

*For Hospitals, Asylums, Infirmaries, Dispensaries, Medical Practitioners, Students, Nurses, and the Charitable Public.*

Vol. IX.—No. 216.] [November 15, 1890.

### Diphtheria at Public Schools.

When head masters have as firm a grasp of things as they now have of words, diphtheria at public schools will be like the snakes of Iceland—it will not exist. There are few more convincing proofs of the precedence of mind in the order of existence than the devotion of the cultivated intellect to words and notions, and its comparative indifference to concrete facts. So decisively is this the case that even the physical sciences, such as chemistry and biology, become converted into sciences of mere words and propositions as they are studied at public schools. A young man would no more think of applying the knowledge of physiology acquired at Eton or Winchester to the prevention of an attack of pleurisy in the hunting-field than he would think of calling a groom to tighten his saddle girths in the tongue of Homer. But this is stupid; it shows a real want of intellect. Five hundred years hence, or sooner we may hope, educated men will not be childishly ignorant of the hard, practical worth of elementary scientific facts, any more than head masters and senior boys are now ignorant of the difference in value between a penny and a pound. For hundreds of years words and notions have been not only the subject matter, but the very life pupose of public schools; and even now, when science is professedly taught, it is taught as furnishing additional matter for new words and new notions, that have in them a certain value for purposes of mental discipline. This we owe to the scholastic philosophy, and even to a large extent to Aristotle. But new times demand new knowledge and new practice; and whilst we need not love Aristotle less, we must learn to love Bacon more. Until the physical sciences are handled in the school exactly as the steak pie is handled by the intelligent cook in the kitchen, they are little better than useless. They are only formulæ, and, still more formulæ, effectually divorced from facts. Is there any man in the position of a head master so dull as not to see this? Mental discipline is a thing of prime worth; but such a knowledge of things and of the facts of science in general as can be used in daily practice is of a value which is only second to that of mental discipline itself. Head masters will do well to suffer the word of exhortation on this point. Until they grasp something of the importance of the elementary principles here set forth, they cannot do their duty to the boys under their charge or to the parents who give into their hands for a series of years their dearest and most precious possessions.

According to a morning contemporary, "The Rev. G. Bell, Master of Marlborough College, writing to the *Marlborough Times*, states that the five diphtheria patients are all convalescent, and two have left the sanatorium and gone home. No case has occurred since October 17th, and the school is at present in its usual healthy condition. Dr. Parkes, who has paid a second visit to the College, states that there is the very strongest probability that the outbreak was due to personal infection from an unrecognisable case introduced into the school at the commencement of the term."

Now this case of Marlborough offers a text from which the whole subject of diphtheria, and of course typhoid, may be profitably discussed. In the first place, here is an outbreak of the former disease; and five boys are attacked almost simultaneously. Everybody, from the head master downwards, is thrown into a state of alarm. The school doctor is called in, and treats the cases, but cannot be at all certain of their origin. Naturally the drains are thought of, and examined. By way of supporting the school doctor, and saving the reputation of the College, an expert is sent for. He, no doubt, examines the drains with more or less thoroughness, and finds nothing which he can certainly fix upon as the undoubted origin of the mischief. Thereupon he writes his report. "An unrecognisable case introduced into the school at the commencement of the term." That is the probable, nay, almost certain, source of the mischief. How can the school help that? The school, indeed, is the victim of a misfortune, and demands our sympathies accordingly.

It is the old familiar story—too old and much too familiar to satisfy any minds except those that desire to be satisfied on the easiest possible terms. Dr. Parkes bears an honoured name, and the true explanation may be that which he suggests. But, as we have pointed out, it is the explanation which is almost invariably given in such cases; and we frankly confess our scepticism. However it may be at Marlborough in the present instance, there is no doubt at all that the usual cause of an outbreak of diphtheria is some poison generated in the immediate locality, on the spot in fact, and affecting simultaneously, or almost simultaneously, a great number of persons who are exposed to it. Diphtheria certainly is not a markedly infectious disease. On the contrary, if the sanitary conditions be even moderately good, it is one of the most easily stamped out of all the diseases to which infection attaches. The commonest by far of all causes of diphtheria is a poison in the air, or in the food or drink, but especially in the air. This will account for at least ninety per cent. of all the cases of the disease. Its transmission from person to person will not account for nine per cent. If this be so, and it undoubtedly is, we venture to suggest to the authorities at Marlborough that they should by no means be content until the whole sanitary condition of the college buildings has been thoroughly investigated, and until every inch of drainage, large and small, has been examined personally, either by the head master or by some practical man on whose integrity and capacity he can thoroughly rely. There is nothing profoundly mysterious about drainage; and if the head master is as practical a person as he ought to be, he will not let his mind be at rest until he is as familiar with the details of the school drainage as he is with the verbs in his Latin Delectus.

So much for Marlborough. On the general subject of public schools it is not too much to say that every school which is fortunate enough to be placed in the country ought to be as free from diphtheria and typhoid fever as it is from leprosy. If half as much money had been spent in the making of intelligent sanitary arrangements as has been spent in the building and adorning of the college chapel at most of the public schools, those diseases would not have been known there. To begin with, there should not be a single water-closet or sink drain in the whole school buildings. All the closets should be distant at least a hundred yards from the dormitories and living and teaching apartments. They should be abundantly numerous, and should be placed under the charge of proper attendants, as at railway stations. The cesspool system should be adopted; the branch drains and the trunk drain should all be thoroughly ventilated; and the cesspools should also be ventilated, emptied and disinfected, and their contents carried to the soil once a week. This is a rational system, and as simple and easy of application as the Rule of Three. Its simplicity, indeed, in the eyes of men of sense, is its chief recommendation. Of course we have here given but the barest outline of the system. A full description, however, will be found in "Helps to Health" (Kegan Paul, Trench, and Co.), p. 135. Is it objected that it would be costly? Not at all. The initial cost would be very little compared with the capitalised value of the school buildings and endowments, and the annual expense of maintenance would be a mere fraction of the whole school revenues. This simple and effective system might be applied to every public school in the country within two years. If the minds of head masters and school managers develop in a scientific direction as slowly in the future as they have done in the past, it will probably take two hundred to get it accomplished. We English are a fearful and wonderful race.

